# Comparative In Vitro Response of HaCaT and A375 Melanoma Cells to Zinc Oxide Nanoparticles Synthesized Using Puerarin

**DOI:** 10.3390/jox16040146

**Published:** 2026-08-04

**Authors:** Sergio Liga, Raluca Vodă, Lavinia Lupa, Raluca Pop, Diana Haj Ali, Iasmina-Alexandra Predescu, Cristina Adriana Dehelean, Francisc Péter

**Affiliations:** 1 Faculty of Chemical Engineering, Biotechnologies and Environmental Protection, Politehnica University Timișoara, Vasile Pârvan No. 6, 300223 Timișoara, Romania; sergio.liga96@gmail.com (S.L.); lavinia.lupa@upt.ro (L.L.); francisc.peter@upt.ro (F.P.); 2 Faculty of Pharmacy, “Victor Babeș” University of Medicine and Pharmacy Timișoara, Eftimie Murgu Square No. 2, 300041 Timișoara, Romania; pop.raluca@umft.ro; 3 Doctoral School, “Victor Babeș” University of Medicine and Pharmacy Timișoara, Eftimie Murgu Square No. 2, 300041 Timișoara, Romania; iasmina-alexandra.predescu@umft.ro; 4 University Clinic of Toxicology, Drug Industry, Management and Legislation, Faculty of Pharmacy, “Victor Babeș” University of Medicine and Pharmacy Timișoara, Eftimie Murgu Square No. 2, 300041 Timișoara, Romania; cadehelean@umft.ro; 5 Research Center for Pharmaco-Toxicological Evaluation, “Victor Babeș” University of Medicine and Pharmacy Timișoara, Eftimie Murgu Square No. 2, 300041 Timișoara, Romania; 6 Renewable Energy Research Institute—ICER, Politehnica University Timișoara, Gavril Musicescu Street No. 138, 300501 Timișoara, Romania

**Keywords:** Puerarin, zinc oxide nanoparticles, nanobiocomposites, A375 malignant melanoma cells, cytocompatibility, clonogenic assay

## Abstract

Zinc oxide nanobiocomposites were successfully synthesized using a green synthesis approach based on the isoflavone Puerarin, resulting in the formation of PUE-ZnO NPs. Building on their previous physicochemical and in ovo characterization, the present study aimed to comparatively evaluate the biological response induced by PUE-ZnO NPs in non-tumorigenic HaCaT keratinocytes and A375 melanoma cells. Cell viability was assessed by MTT assay, and the cellular response was further investigated through bright-field morphological evaluation, Hoechst 33342 nuclear staining, MitoTracker mitochondrial staining, and clonogenic assay. PUE-ZnO NPs showed a favorable cytocompatibility profile in HaCaT cells, with viability remaining above 80% at the highest tested concentration and only moderate impairment of clonogenic capacity at higher concentrations. In contrast, A375 melanoma cells exhibited a stronger response, characterized by reduced viability, marked morphological alterations, nuclear changes, mitochondrial staining disruption, and a pronounced decrease in colony-forming ability. PUE-ZnO NPs may represent a promising phytochemical-assisted ZnO-based nanosystem for further investigation in melanoma-related models.

## 1. Introduction

Metal oxide nanoparticles are emerging biomedical materials, with multiple utilizations in tissue therapy, immunotherapy, diagnosis, dentistry, regenerative medicine, wound healing and biosensing platforms [1]. Accordingly, there is a constantly growing research effort focused on novel metal oxide nanomaterials, trying to find the right balance between their positive therapeutic effects and possible toxic side effects. The green synthesis of metal oxide nanoparticles involving plant extracts or plant components turned out to be not only a cost-effective and eco-friendly method but also provided new nanobiocomposites which can benefit from the well-known pharmaceutical effects of the phytochemical constituents of the employed plants, particularly of flavonoids [2,3,4]. Among metal oxide nanomaterials, zinc oxide nanoparticles (ZnO NPs) have attracted considerable scientific interest because of their distinctive physicochemical properties, high surface area, photostability, cost-effective production, and potential for functionalization [5]. These characteristics have supported their investigation in antimicrobial treatments, cancer therapy, drug-delivery systems, bioimaging, tissue engineering, and other biomedical applications [5,6,7].

The biomedical relevance of ZnO NPs is partly associated with their broad antimicrobial activity and their ability to interact with microbial surfaces, release Zn^2+^ ions, and promote the generation of reactive oxygen species [6,8]. The resulting oxidative stress may damage membrane lipids, proteins, and nucleic acids, disrupt essential cellular processes, and ultimately reduce microbial survival [8].

Beyond their antimicrobial properties, ZnO NPs have received increasing attention as potential anticancer agents and as nanoplatforms for the delivery of therapeutic compounds [9,10]. Their anticancer effects have primarily been associated with intracellular Zn^2+^ accumulation, excessive reactive oxygen species generation, disruption of cellular redox homeostasis, mitochondrial dysfunction, and activation of apoptotic pathways [6,8]. Following cellular uptake, ZnO NPs may accumulate within endosomal and lysosomal compartments, where acidic conditions can promote nanoparticle dissolution and increase the intracellular availability of Zn^2+^ ions [6,10]. Increased oxidative stress may subsequently decrease mitochondrial membrane potential, promote the release of cytochrome c, alter the balance between pro- and anti-apoptotic proteins, and activate initiator and executioner caspases [6,9]. ZnO NP exposure has also been associated with cell-cycle alterations, lysosomal damage, autophagosome accumulation, impaired autophagic flux, and the accumulation of damaged mitochondria, although the contribution of these mechanisms varies among experimental models [6,8].

Nevertheless, the clinical translation of ZnO-based systems remains limited by unresolved questions concerning long-term toxicity, stability, reproducibility, standardized physicochemical characterization, scalable production, and comprehensive safety evaluation [5,10]. ZnO NPs have demonstrated biological activity in experimental models of cutaneous malignancy, including human A375 melanoma cells [11]. HaCaT keratinocytes and A375 melanoma cells represent distinct cutaneous cell lineages, as keratinocytes and melanocytes have different biological origins and functions [12]. Consequently, differences observed between these models should be interpreted as differential susceptibility between a keratinocyte model and a melanoma model rather than as definitive evidence of universal cancer-cell specificity [12,13]. Nevertheless, exposing both cell lines to the same nanoparticle preparation, concentration range, exposure periods, and analytical procedures can provide relevant information regarding relative cytotoxicity and a possible preferential effect against the melanoma model [9,13]. Such a comparative approach may contribute to determining whether the biological activity of the investigated ZnO NPs is accompanied by a comparatively lower impact on keratinocytes and may help identifying cellular mechanisms associated with the different responses of the two models [6,9].

In our previous study [14], zinc oxide nanobiocomposites (abbreviated PUE-ZnO NPs) were obtained through a green synthesis route in which the isoflavone Puerarin (abbreviated PUE) mediated nanoparticle formation, contributed to stabilization through its carbonyl and hydroxyl functional groups, and remained attached itself to the nanoparticles. The presence of PUE-derived functional groups in the final nanobiocomposite was supported by ATR-FTIR analysis, while XRD confirmed the formation of a crystalline zincite structure without detectable impurity peaks. SEM and AFM analyses revealed a predominantly quasi-spherical, rough, and agglomerated morphology, although the dimensional estimates varied according to the characterization method employed. The resulting PUE-ZnO NPs exhibited antimicrobial activity against *Staphylococcus aureus*, *Streptococcus pyogenes*, *Escherichia coli*, and *Candida parapsilosis*, whereas no inhibitory concentration was established for *Pseudomonas aeruginosa* under the investigated conditions. At a concentration of 100 µg/mL, the PUE-ZnO NPs were classified as non-irritant in the HET-CAM assay and did not induce relevant alterations in the normal developing CAM vasculature after 24 h of exposure, indicating good vascular tolerance under the tested experimental conditions.

Collectively, these findings provided a rationale for the further biological evaluation of PUE-ZnO NPs in cutaneous cellular models. Therefore, the present study aimed to comparatively assess their effects on immortalized human keratinocytes (HaCaT) and A375 human melanoma cells, with the objective of determining whether PUE-ZnO NPs exhibit preferential cytotoxicity toward the melanoma model.

## 2. Materials and Methods

### 2.1. Reagents and Instruments

Dulbecco’s Modified Eagle’s Medium (DMEM), fetal bovine serum (FBS), and penicillin/streptomycin solution (100 U/mL–100 μg/mL) used for cell culture were obtained from PAN-Biotech GmbH (Aidenbach, Germany). Phosphate-buffered saline (PBS) and sodium lauryl sulfate (SLS) were purchased from Sigma-Aldrich (Merck KGaA, Darmstadt, Germany). Cell viability was assessed using the MTT Cell Proliferation Assay Kit [3-(4,5-dimethylthiazol-2-yl)-2,5-diphenyltetrazolium bromide] supplied by Roche Diagnostics (UK). For fluorescence-based analyses, Hoechst 33342 and MitoTracker™ Red CMXRos were acquired from ThermoFisher Scientific (Waltham, MA, USA). A ready to use 4% paraformaldehyde solution prepared in PBS was purchased from Santa Cruz Biotechnology (Dallas, TX, USA), while a 1% crystal violet solution used for colony staining was obtained from Electron Microscopy Sciences (Hatfield, PA, USA).

The in vitro analyses were performed using the Lionheart™ FX automated microscope, the Cytation™ 5 multimode microplate reader, and Gen5™ Microplate Data Collection and Analysis Software version 3.14 (BioTek Instruments Inc., Winooski, VT, USA).

### 2.2. In Vitro Experimental Methodology

#### 2.2.1. Cell Lines and Culture Conditions

The HaCaT (300493) immortalized human keratinocyte cell line, obtained from CLS (Eppelheim, Germany), and human melanoma cell line A375 (CRL-1619™) purchased from ATCC (Manassas, VA, USA) were cultured in Dulbecco’s Modified Eagle’s Medium (DMEM) supplemented with 10% fetal bovine serum (FBS) and 1% penicillin–streptomycin and maintained in a humidified incubator at 37 °C in a 5% CO2 atmosphere. The use of the 1% penicillin/streptomycin combination is justified to reduce the risk of bacterial contamination, a standard method recommended by the manufacturer. Penicillin is a β-lactam antibiotic with predominant activity against Gram-positive bacteria, while Streptomycin belongs to the aminoglycoside class and is effective against both Gram-positive and Gram-negative bacteria. The combination of these two antibiotics is frequently used due to their synergistic effect which contributes to increased antibacterial efficacy [15,16].

The PUE-ZnO NPs were dispersed in complete culture medium to obtain a 50 mg/mL stock solution. Subsequently, working solutions with final concentrations of 25, 50, 75, 100, and 200 µg/mL were prepared from the stock solution, and HaCaT and A375 cells were exposed to these concentrations for 24 h. The selected concentrations were based on previously published in vitro studies that evaluated the substance’s anticancer activity in melanoma cells, chosen to cover a biologically relevant range previously reported in the literature [17,18].

#### 2.2.2. Cell Viability Assessment Through MTT Assay

The safety profile and the cytotoxic effect of PUE-ZnO NPs on A375 and HaCaT cells were examined using the MTT (3-(4,5-dimethylthiazol-2-yl)-2,5-diphenyltetrazolium bromide) assay. After 24 h of exposure to the sample at concentrations (25–200 μg/mL), the culture medium was replaced with 100 µL of fresh medium supplemented with 10 µL of MTT reagent in each well, followed by incubation for 3 h under standard cell culture conditions. Then, 100 µL of MTT solubilization solution was added to dissolve the formazan crystals, and the plate was left at room temperature for 30 min. Cell viability was then determined by measuring absorbance at 570 nm, with 630 nm used as the reference wavelength, using a Cytation 5 microplate reader.

#### 2.2.3. Bright-Field Evaluation of Cell Morphology

Changes in cell morphology upon treatment with PUE-ZnO NPs were evaluated in A375 and HaCaT cells. Both cell lines were seeded into 96-well plates at a density of 1 × 10^4^ cells per well and incubated with treatment at concentrations ranging from 25 to 200 µg/mL for 24 h. After the incubation period, representative images were taken using a Lionheart FX and analyzed and processed using Gen5™ Microplate Data Collection and Analysis software in version 3.14.

#### 2.2.4. Evaluation of Mitochondrial and Nuclear Alterations

Nuclear and mitochondrial morphology in HaCaT and A375 cells following exposure to PUE-ZnO NPs was examined by combining Hoechst 33342 nuclear staining with MitoTracker Red CMXRos mitochondrial labeling. Cells were seeded in 12-well culture plates at a density of 1 × 10^5^ cells per well and maintained under standard culture conditions until reaching the appropriate confluence. Cells were subsequently exposed to the sample at concentrations of 25–200 µg/mL for 24 h.

To visualize the mitochondrial network, a MitoTracker Red CMXRos working solution was prepared by diluting the 1 mM stock solution in complete culture medium to a final concentration of 300 nM. Following treatment, the staining solution was added to the cells, which were incubated for 30–45 min at 37 °C. Excess dye was removed by carefully washing the cells with PBS, after which fixation was performed using 4% paraformaldehyde for 10 min at room temperature. The fixed cells were rinsed once more with PBS before proceeding to nuclear staining. For nuclei labeling of HaCaT and A375 cells, Hoechst 33342 was used in a working solution prepared by diluting the stock reagent 1:2000 in PBS. After incubation in the dark for 5–10 min, the staining solution was discarded, and the cells were washed three times with PBS to remove unbound dye.

Fluorescence images were captured with a Lionheart FX automated microscope using a 20× objective, while image acquisition and analysis were performed using Gen5™ Microplate Data Collection and Analysis Software in version 3.14. The experimental protocol was based on the method reported by Vicaș et al. [19]. In addition, using the following formula, the apoptotic index (AI) was calculated as follows (Equation (1)):

(1) $$\mathrm{AI}\;(\%)\;=\;\frac{Number\;of\;apoptotic\;cells}{Total\;number\;of\;cells}\;$$

#### 2.2.5. Colony Formation Assay

The colony formation assay was performed using a protocol adapted from the methodology previously reported by Smeu et al. [20]. HaCaT and A375 cells were seeded into 96-well plates at a density of 100 cells per well and allowed to adhere before treatment. Cells were subsequently exposed to PUE-ZnO NPs (25–200 µg/mL). After 24 h of treatment, the culture medium was replaced with fresh medium and renewed periodically during the subsequent 10-day incubation period to support colony development. After the incubation period, the colonies were fixed with 4% paraformaldehyde for 10 min at room temperature and rinsed with PBS. Colony staining was performed using a 0.2% crystal violet solution prepared in PBS for 10 min, after which excess dye was removed by repeated washing with distilled water. The bound stain was solubilized with 1% SLS. Images of the resulting colonies were captured using a Lionheart FX imaging system, while absorbance was recorded at 550 nm using a Cytation 5 microplate reader. Quantitative image analysis was performed using Gen5 software (version 3.14).

Colony formation was quantified according to the method described by Han et al. [21]. Following crystal violet staining, the dye was solubilized using SLS, and the absorbance was measured at 550 nm using the Cytation 5 multimode reader. The colony formation rate was then calculated by dividing the absorbance value of each treated group by that of the untreated control group. The uncropped images used in the manuscript are provided in Supplementary Materials.

### 2.3. Statistical Analysis

Statistical analyses were carried out using GraphPad Prism software (version 11.0.2; GraphPad Software, San Diego, CA, USA). Differences between treated samples and the untreated control group were evaluated by one-way analysis of variance (ANOVA), followed by Dunnett’s post hoc multiple comparison test. Statistical significance was expressed as follows: * *p* < 0.05; ** *p* < 0.01; *** *p* < 0.001; **** *p* < 0.0001. All assays were performed in biological and technical triplicates.

## 3. Results

The synthesis of ZnO nanoparticles with PUE, an isoflavone with increasingly growing biomedical interest [21,22], was accomplished as described in our previous work [14]. Since the comprehensive physicochemical characterization of these nanoparticles was also done before, our attention turned to the demonstration of a selective cytotoxic effect against tumoral cells.

### 3.1. In Vitro Experimental

#### 3.1.1. Cell Viability Assessment Following PUE-ZnO NP Treatment in A375 and HaCaT Cells

The first step in the biological evaluation of PUE-ZnO NPs consisted of assessing their effects on cell viability using the MTT assay following 24 h of exposure to concentrations ranging from 25 to 200 μg/mL.

**Figure 1:**
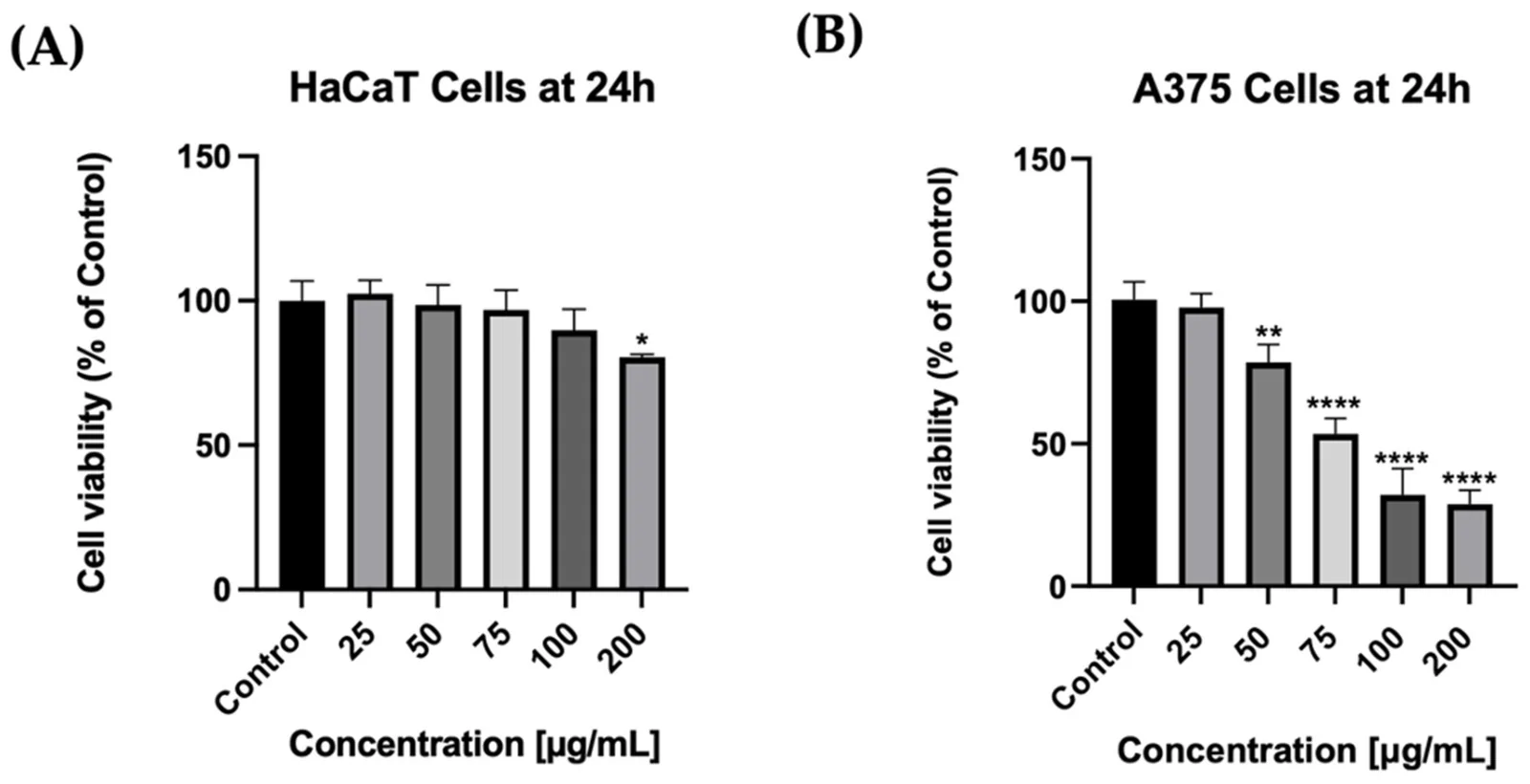
Cell viability following 24 h exposure to PUE-ZnO NPs at concentrations ranging from 25 to 200 µg/mL in (**A**) HaCaT and (**B**) A375 cells. The results are expressed as percentages (%) normalised to the untreated control group. Data are presented as mean values ± standard deviation (SD) from three independent experiments, each performed in triplicate. Statistical differences between the treated groups and the control were assessed using one-way ANOVA followed by Dunnett’s multiple comparisons post hoc test (* *p* < 0.05; ** *p* < 0.01; **** *p* < 0.0001).

As shown in Figure 1A, PUE-ZnO NPs exhibited good cytocompatibility at lower concentrations, with cell viability remaining above 95%, at 102.39%, 98.54%, and 96.85% at 25, 50, and 75 μg/mL, respectively. At higher concentrations of 100 and 200 μg/mL, a decrease in viability to 89.86% and 80.44% was observed. However, these results indicate that PUE-ZnO NPs exhibit a favorable biocompatibility profile on human HaCaT keratinocytes. In A375 cells, according to Figure 1B, PUE-ZnO NPs induced a dose-dependent reduction in viability, reaching 56.49%, 32.05%, and 28.86% at 75, 100, and 200 μg/mL, respectively, indicating a marked cytotoxic effect at higher concentrations.

#### 3.1.2. Evaluation of Cellular Morphology Following PUE-ZnO NP Treatment in A375 and HaCaT Cells

The next step in the biological evaluation of PUE-ZnO NPs involved microscopic analysis of cell morphology, which provides important information regarding cell integrity, their growth capacity, and potential changes induced by the treatment.

**Figure 2:**
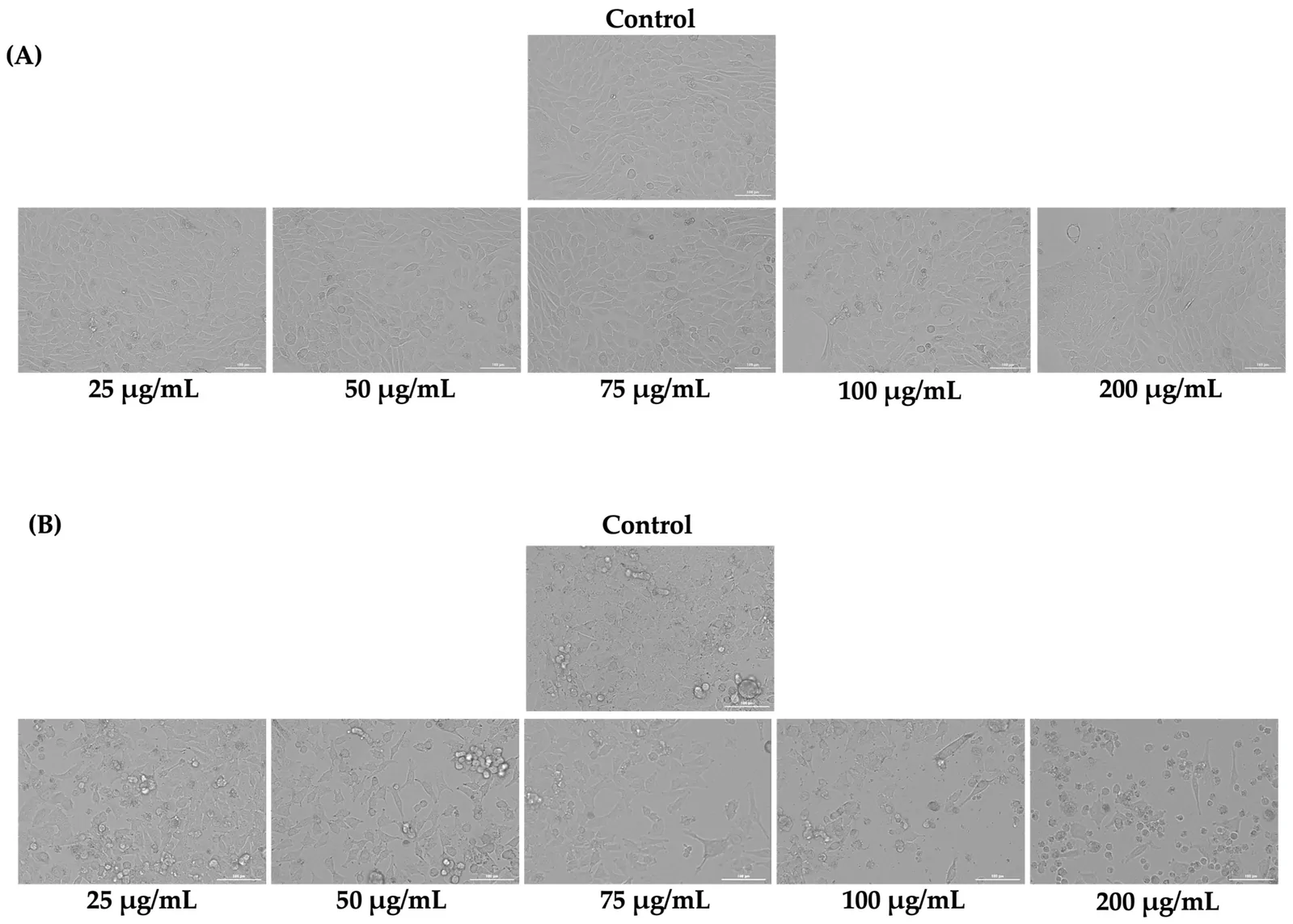
Representative images illustrating the morphological changes observed in (**A**) HaCaT and (**B**) A375 cells after 24 h of treatment with PUE-ZnO NPs at concentrations ranging from 25 to 200 µg/mL. The scale bar indicates 100 µm.

As shown in Figure 2A, treatment with PUE-ZnO NPs did not induce noticeable morphological alterations in HaCaT cells. The cells retained their characteristic morphology throughout the tested concentration range, while only a slight decrease in cell confluence was observed. In contrast, exposure of A375 cells to PUE-ZnO NPs (25–200 µg/mL) resulted in evident morphological changes, which became more pronounced at 100 and 200 µg/mL (Figure 2B). These alterations were characterized by massive cell rounding, loss of the typical morphology, and cellular fragmentation. Furthermore, a concentration-dependent reduction in cell confluence was observed across all tested concentrations.

#### 3.1.3. Evaluation of Nuclear and Mitochondrial Morphology Following PUE-ZnO NP Treatment in A375 and HaCaT Cells

To assess the effects of the tested substances on nuclear and mitochondrial architecture, fluorescence imaging was performed using Hoechst staining for nuclear morphology and MitoTracker for mitochondrial visualization.

**Figure 3:**
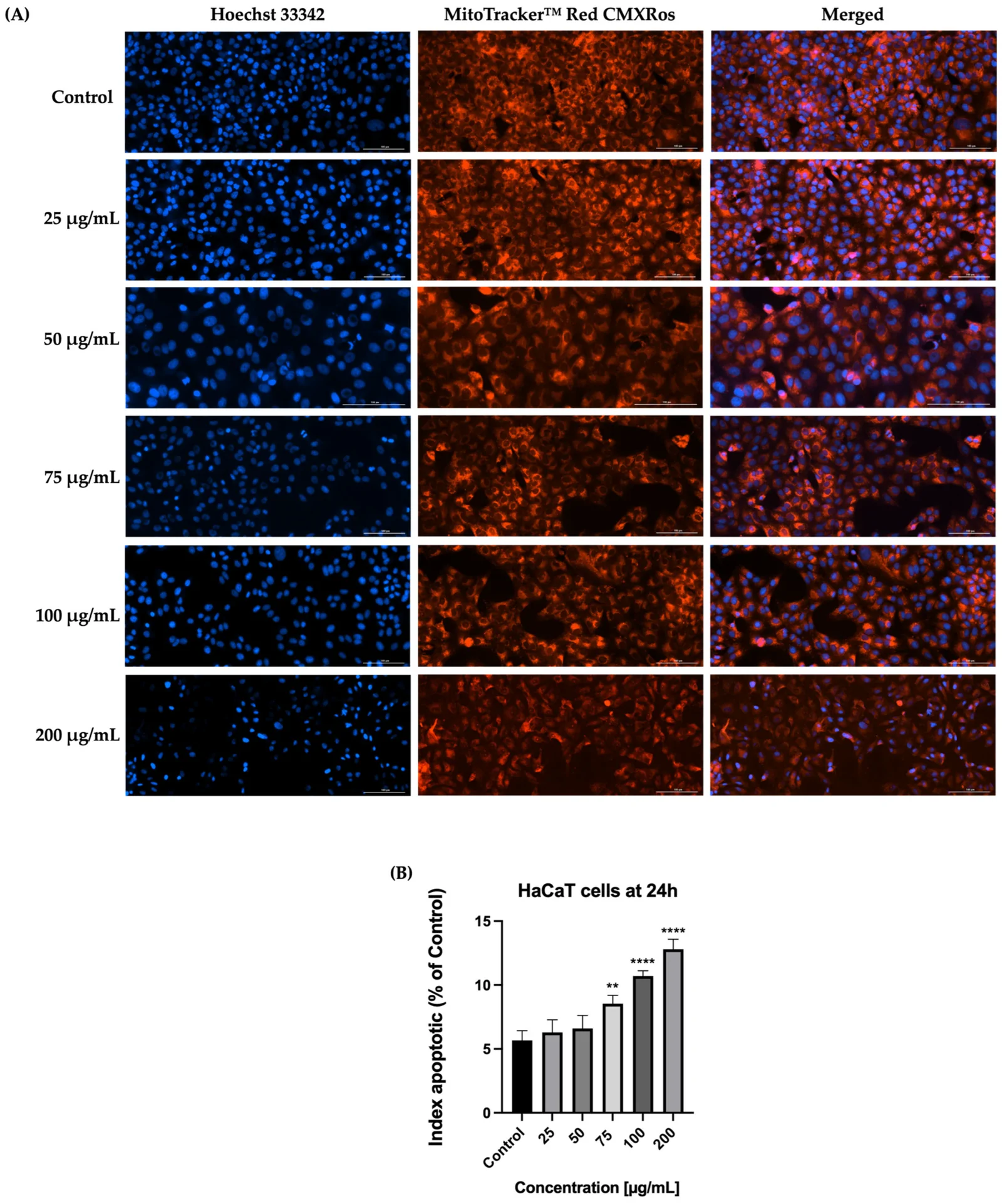
The effect of treatment with PUE-ZnO NPs at concentrations of 25–200 µg/mL on HaCaT cells, assessed by nuclear and mitochondrial staining after 24 h of exposure: (**A**) representative fluorescence microscopy images showing nuclear and mitochondrial morphology; (**B**) graphical representation of the apoptotic index. Data are shown as mean ± standard deviation (SD) from three independent experiments and are expressed relative to the untreated control group, which was assigned a value of 100%. Statistical comparisons between treated and control cells were performed using one-way ANOVA followed by Dunnett’s post hoc multiple-comparison test. Statistical significance is denoted as follows: ** *p* < 0.01; **** *p* < 0.0001. The scale bar indicates 100 µm.

In the case of the HaCaT cell line, the evaluation of nuclear morphology revealed, overall, an appearance similar to that of the control group. However, at the highest concentration tested (200 µg/mL), slight changes were observed, such as mild chromatin condensation and the rounding of nuclei. Regarding mitochondrial morphology, the mitochondrial architecture remained comparable to that of the control cells, with only a slight condensation of the mitochondrial signal observed at a concentration of 200 µg/mL (Figure 3A). The apoptotic index was used to quantify the nuclear alterations observed following treatment (Figure 3B). In the untreated control group, the apoptotic index was 5.67%. Following exposure to PUE-ZnO NPs, this parameter increased progressively to 6.30%, 6.61%, 8.54%, 10.70%, and 12.81% at 25, 50, 75, 100, and 200 µg/mL, respectively.

**Figure 4:**
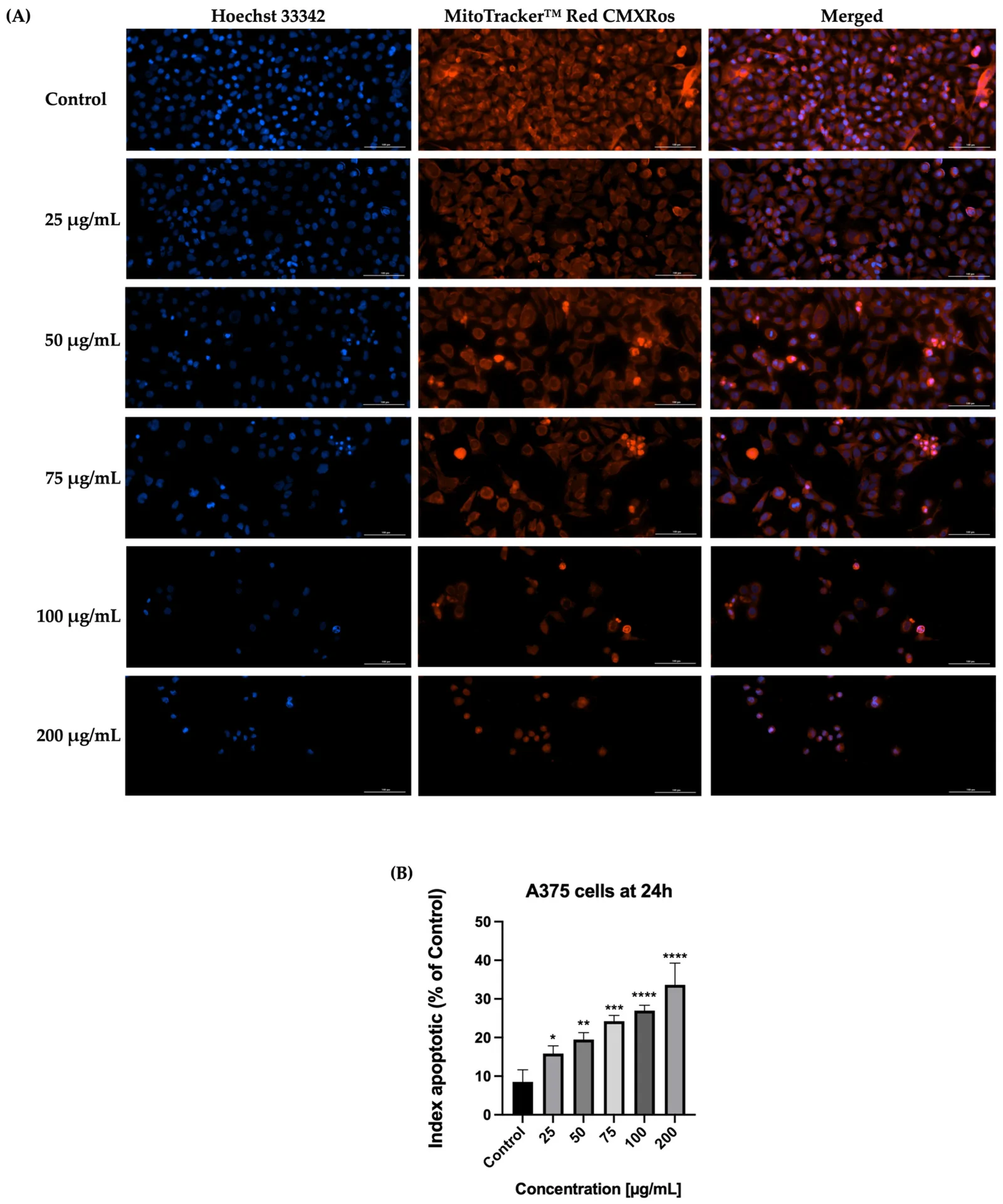
The effect of treatment with PUE-ZnO NPs at concentrations of 25–-200 µg/mL on A375 cells, assessed by nuclear and mitochondrial staining after 24 h of exposure: (**A**) representative fluorescence microscopy images showing nuclear and mitochondrial morphology; (**B**) graphical representation of the apoptotic index. Data are shown as mean ± SD from three independent experiments and are expressed relative to the untreated control group, which was assigned a value of 100%. Statistical comparisons between treated and control cells were performed using one-way ANOVA followed by Dunnett’s post hoc multiple-comparison test. Statistical significance is denoted as follows: * *p* < 0.05; ** *p* < 0.01; *** *p* < 0.001; **** *p* < 0.0001. The scale bar indicates 100 µm.

Regarding the impact on A375 cells according to Figure 4A, the nuclear and mitochondrial morphology at 25 µg/mL appeared similar to the control, although a slight reduction in confluence was noticeable. The overall appearance of both nuclei and mitochondria remained largely unchanged at this concentration. Beginning at 50 µg/mL, chromatin condensation became evident, accompanied by early mitochondrial alterations, including a tendency of mitochondria to adopt a more rounded morphology. At concentrations of 100–200 µg/mL, structural disruption became pronounced, with mitochondria displaying a distinctly retracted, rounded appearance and nuclei exhibiting marked condensation and noticeable changes in shape. Nuclear changes were also quantified using the apoptotic index, which showed a concentration-dependent increase (Figure 4B). While the control group presented an apoptotic index of 8.53%, treatment led to values of 15.84%, 19.49%, 24.23%, 26.99%, and 33.67%, respectively, demonstrating a progressive increase in the number of apoptotic cells.

#### 3.1.4. Evaluation of Colony Formation Following PUE-ZnO NP Treatment in HaCaT and A375 Cells

Beyond evaluating the potential of PUE-ZnO NPs to induce acute cytotoxicity following a 24-h treatment, its effects on long-term cell growth and proliferation were also investigated in both HaCaT and A375 cell lines using the colony formation assay after 10 days. This assay reflects the ability of a single cell to survive, proliferate, and form a colony, representing an important parameter for assessing the long-term impact of a treatment.

**Figure 5:**
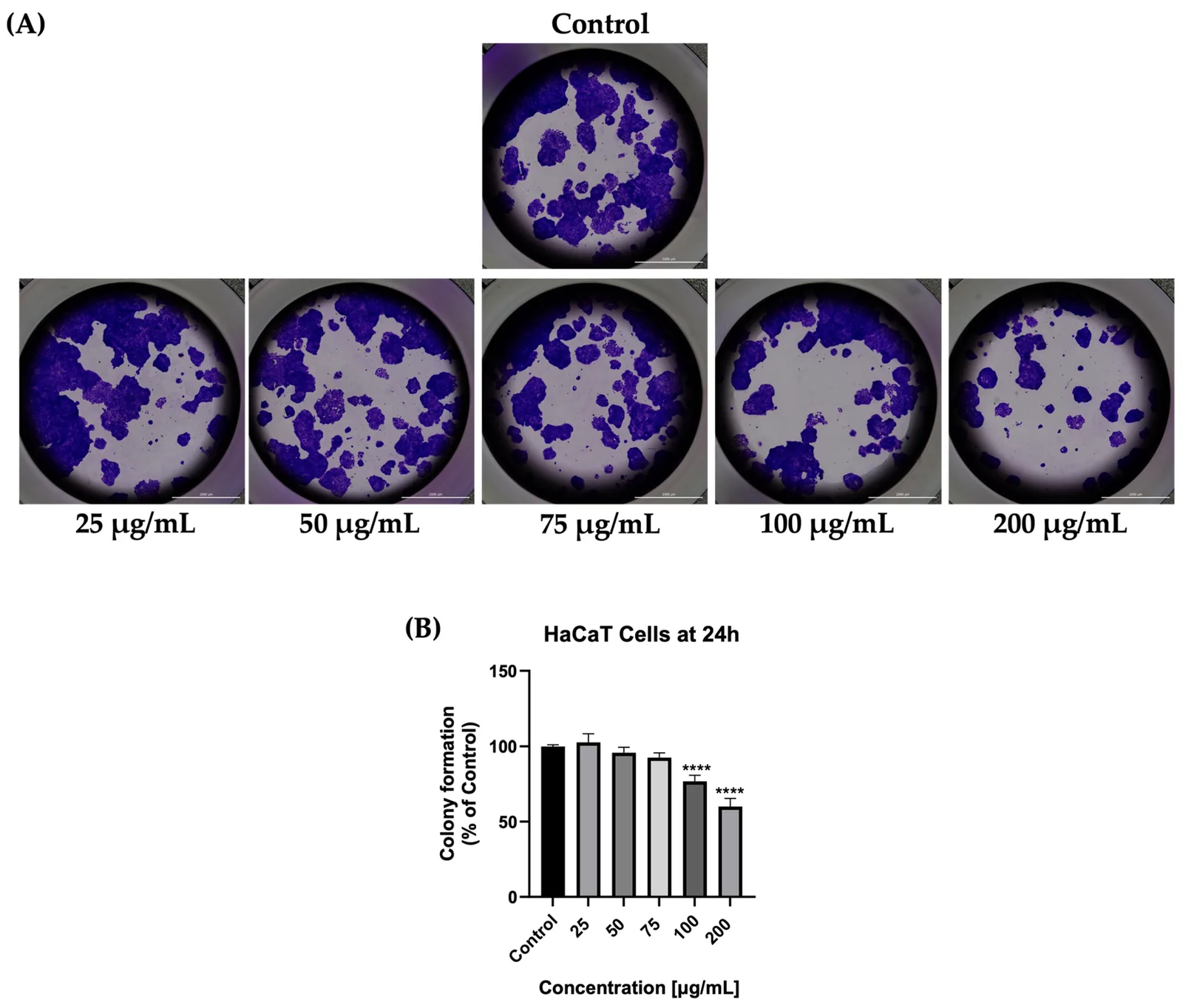
Impact of PUE-ZnO NPs (25–200 µg/mL) on colony formation in HaCaT cells following 24 h of exposure: (**A**) representative images showing colony formation following 24 h treatment with PUE-ZnO NPs. The scale bar represents 2000 µm; (**B**) quantification of colony formation after 24 h exposure to PUE-ZnO NPs, expressed as a percentage relative to the untreated control group (100%). Data are presented as mean ± standard deviation (SD). Statistical differences compared to the control were analyzed using one-way ANOVA followed by Dunnett’s post hoc test; **** *p* < 0.0001 compared to the control group.

In HaCaT cells, microscopic evaluation (Figure 5A) suggested a slight reduction in the number and/or size of colonies, which became more evident only at the highest tested concentrations (100 and 200 μg/mL). These observations were further confirmed by quantitative analysis (Figure 5B), which revealed the following colony formation capacity values relative to the untreated control: 102.73%, 95.87%, 92.56%, 76.82%, and 60.03% for concentrations of 25, 50, 75, 100, and 200 μg/mL, respectively.

**Figure 6:**
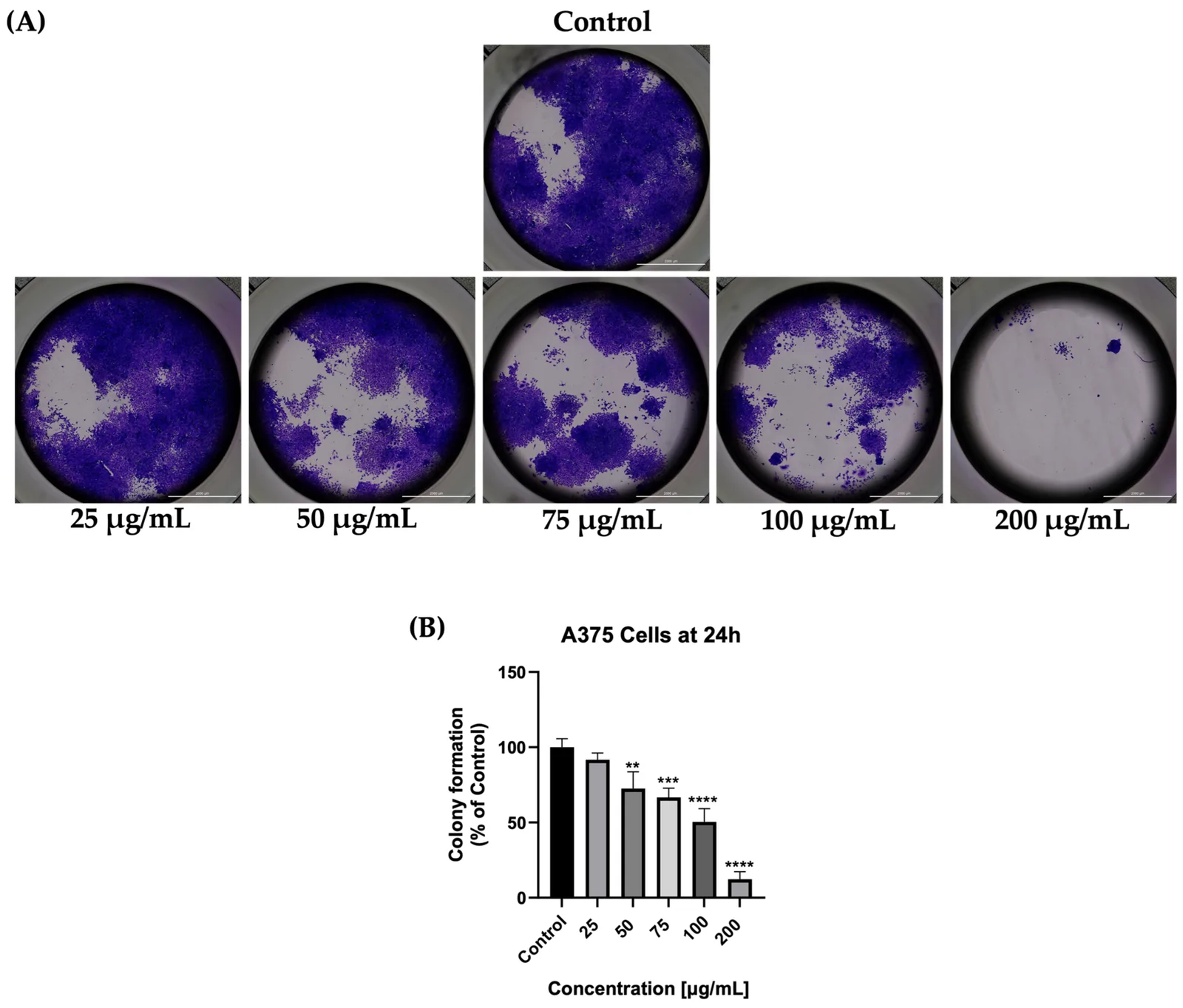
Impact of PUE-ZnO NPs (25–200 µg/mL) on colony formation in A375 cells following 24 h of exposure: (**A**) representative images showing colony formation following 24 h treatment with PUE-ZnO NPs. The scale bar represents 2000 µm; (**B**) quantification of colony formation after 24 h exposure to PUE-ZnO NPs, expressed as a percentage relative to the untreated control group (100%). Data are presented as mean ± standard deviation (SD). Statistical differences compared to the control were analyzed using one-way ANOVA followed by Dunnett’s post hoc test; ** *p* < 0.01; *** *p* < 0.001; **** *p* < 0.0001 compared to the control group.

Regarding the A375 cell line, PUE-ZnO NPs induced a concentration-dependent reduction in the colony-forming ability compared to the untreated control group, affecting both the number and size of the colonies (Figure 6A). Quantitative analysis (Figure 6B) confirmed a progressive decrease in clonogenic capacity with increasing concentration, reaching 91.72%, 72.54%, 66.72%, 50.43%, and 12.26% at 25, 50, 75, 100, and 200 µg/mL, respectively. These findings indicate that PUE-ZnO NPs markedly impair the long-term proliferative capacity of A375 melanoma cells, particularly at higher concentrations.

## 4. Discussion

Melanoma is an aggressive cutaneous malignancy arising from the malignant transformation of melanocytes and remains a major clinical challenge due to its high metastatic potential and melanoma-related mortality, and its development is associated with several risk factors (e.g., ultraviolet radiation exposure, family history, genetic predisposition [23]. In recent years, natural compounds have attracted increasing interest due to their antitumor potential, bioactive properties, and possible use as complementary or adjuvant agents in melanoma-related therapeutic strategies [24]. However, in addition to demonstrating antitumor efficacy against melanoma cells, it is equally important to evaluate their safety profile. Therefore, studies of cytocompatibility using normal skin cell models are an essential component of preclinical evaluation. Moreover, the progression of melanoma is also influenced by the skin microenvironment. Keratinocytes, which form the outer layer of the epidermis and are in direct contact with melanocytes and melanoma cells, are involved in melanogenesis, particularly in the early stages of melanoma development [25].

In this context, HaCaT cells are among the most widely used in vitro models of human keratinocytes because they can be easily cultured and retain several characteristics relevant to the human epidermis. Given the essential role of keratinocytes in skin structure and homeostasis, together with melanocytes and fibroblasts, this cell line is frequently used to assess the cytocompatibility and safety of compounds or formulations intended for dermatological applications [26]. In the present study, HaCaT keratinocytes were used as a non-tumorigenic skin cell model to evaluate the preliminary biocompatibility of PUE-ZnO NPs, while A375 melanoma cells were used as a malignant melanoma model to assess their potential antimelanoma effect. This comparative approach is relevant because an effective dermatological or antimelanoma formulation should ideally reduce melanoma cell viability while maintaining acceptable compatibility with non-tumorigenic skin cells.

Cell viability assays are widely used to evaluate the cytotoxicity or cytocompatibility in vitro of different compounds and formulations. These assays provide information regarding the cellular response after treatment and allow the estimation of cell viability based on specific biological readouts, such as metabolic activity, membrane integrity, or ATP content [27]. The MTT assay is considered the gold standard for assessing cell viability and proliferation and is one of the most widely used methods for evaluating drug toxicity and anticancer activity. It determines cell viability by measuring the enzymatic reduction of tetrazolium into insoluble formazan crystals by metabolically active cells, reflecting the activity of NAD(P)H-dependent oxidoreductases. Its high sensitivity and suitability for high-throughput screening have made it a major advance in cell counting technology. Although other tetrazolium-based assays (e.g., XTT, MTS, WST) are easier to perform because they produce water-soluble formazan, the MTT assay remains one of the most established and widely used methods for evaluating cell viability [28,29]. 

The present study was conceived as a preliminary biological continuation of our previous work, in which PUE was successfully used for the green synthesis of ZnO nanoparticles and was shown to participate in nanoparticle formation and stabilization [14]. In the current investigation, the focus was shifted from green synthesis, physicochemical characterization, antimicrobial activity, angiogenesis modulation, and in ovo tolerability toward a comparative in vitro evaluation of the cellular response induced by PUE-ZnO nanoparticles in HaCaT keratinocytes and A375 melanoma cells. This comparison was intended to assess whether the obtained nanobiocomposite produces a general cytotoxic effect or rather a cell-line-dependent antiproliferative response more pronounced in melanoma cells.

In the present study, the MTT assay was used as an initial screening method to evaluate the cytocompatibility of PUE-ZnO NPs in HaCaT keratinocytes and their cytotoxic potential in A375 melanoma cells. The results obtained in HaCaT cells indicated a favorable cytocompatibility profile, as cell viability remained above 80% even at the highest tested concentration. This observation suggests that PUE-ZnO NPs were well tolerated by keratinocytes under the experimental conditions used. Moreover, the viability values remained above the 70% threshold commonly used for the interpretation of in vitro cytotoxicity according to the ISO 10993-5 standard, which considers a reduction in cell viability greater than 30% as indicative of a cytotoxic effect [30]. Therefore, the response observed in HaCaT cells supports the preliminary biosafety of PUE-ZnO NPs toward a non-tumorigenic keratinocyte model.

Since nanoparticles may interfere with colorimetric viability assays through optical absorption, light scattering, adsorption of assay components, or direct interaction with tetrazolium salts and formazan products, appropriate cell-free nanoparticle controls were included to account for potential assay-related artifacts [31,32,33]. Therefore, appropriate cell-free nanoparticle controls were included in the present study. These controls showed no relevant assay interference, indicating that the observed viability changes reflected biological effects rather than optical or chemical artifacts. Moreover, the MTT data were interpreted together with complementary morphological, nuclear, mitochondrial, and clonogenic assays, which supported the differential response observed between HaCaT and A375 cells. This combined evaluation strengthens the reliability of the results and supports the conclusion that PUE-ZnO NPs induced a more pronounced antiproliferative response in A375 melanoma cells while maintaining acceptable cytocompatibility in HaCaT keratinocytes.

The interpretation of the antiproliferative response should also consider the biological relevance of isoflavone itself. In our previous study on A375 melanoma cells, free PUE was included as a separate comparator and showed a concentration-dependent effect, while its incorporation into a proniosomal gel formulation resulted in a more pronounced reduction in cell viability [17]. Therefore, PUE should not be regarded only as an inert phytochemical involved in nanoparticle synthesis, but also as a bioactive component that may influence the interaction of the PUE-ZnO nanobiocomposite with melanoma cells.

The observed response is consistent with previous studies reporting cell-line-dependent cytotoxicity for phytogenic and flavonoid-associated ZnO nanosystems. Rutin-mediated ZnO nanoparticles showed stronger antiproliferative activity than standard ZnO nanoparticles and free rutin in MCF-7 cells after 48 h [34], while ZnO nanoparticles synthesized using *Limonium pruinosum* extract displayed dose-dependent cytotoxicity against A-431 skin cancer cells, with no detrimental effect on WI-38 normal cells up to 250 µg/mL [35]. Similarly, ZnO nanoparticles obtained from mangosteen peel or water hyacinth extracts inhibited A431 and HaCaT cells more strongly than Vero cells, and their activity was associated with bioactive functional groups present on the nanoparticle surface [36]. In a melanoma-related model, green-synthesized Ag/ZnO nanocomposites obtained using *Pistacia atlantica* leaf extract also produced a dose-dependent cytotoxic response in A375 cells, with comparatively lower toxicity toward normal HDF cells [37]. Although direct comparison among these studies is limited by differences in nanoparticle composition, synthesis route, exposure time, concentration range, and cell models, the available evidence supports the biological relevance of phytochemical-assisted ZnO-based systems as cell-line-dependent anticancer candidates.

The next step in the evaluation of PUE-ZnO NPs was the assessment of cellular morphology using bright-field microscopy. Morphological evaluation is an important component of in vitro cytotoxicity studies, as it allows the visualization of treatment-induced changes such as reduced confluence, cell rounding, detachment, shrinkage, and fragmentation, which may be associated with cellular stress or cell death processes [38]. However, morphological observations alone are not sufficient to clearly discriminate between apoptosis and necrosis and should therefore be interpreted together with complementary assays.

PUE-ZnO NPs did not induce major morphological alterations in HaCaT keratinocytes, except for a reduction in cell confluence at the highest tested concentrations. This observation is consistent with the MTT results, which indicated good cytocompatibility of the formulation in this cell line. In contrast, A375 melanoma cells exhibited marked morphological changes after treatment, including cell rounding, reduced adherence, fragmentation, and decreased cell density, particularly at the highest tested concentrations. These alterations support the cytotoxic effect observed in the MTT assay and suggest that A375 cells are more sensitive to PUE-ZnO NP treatment than HaCaT keratinocytes.

Hoechst 33342 is a cell-permeable fluorescent dye commonly used to assess nuclear morphology and to identify nuclear changes associated with apoptosis, such as chromatin condensation and nuclear fragmentation [39]. In the present study, the evaluation of nuclear morphology showed that PUE-ZnO NPs were well tolerated by HaCaT keratinocytes, with only slight nuclear changes observed at the highest tested concentration. In contrast, A375 melanoma cells displayed progressive nuclear alterations, accompanied by an increase in the apoptotic index, suggesting a concentration-dependent cytotoxic effect of the formulation. Previous studies have reported that exposure of HaCaT cells to ZnO NPs can induce chromatin condensation and nuclear morphology alterations, with more pronounced effects at higher concentrations [40]. Similarly, in the present study, subtle nuclear changes were observed in HaCaT cells only at the highest tested concentration, including slight chromatin condensation and a more rounded nuclear appearance. However, these changes were limited compared with those observed in A375 cells, supporting the higher sensitivity of melanoma cells to PUE-ZnO NP treatment.

Assessing mitochondrial morphology is important in cytotoxicity studies, as mitochondria are involved in energy production, oxidative stress regulation, and apoptosis. MitoTracker fluorescent staining is frequently used to visualize mitochondria in living cells, as these probes can penetrate the cell membrane and accumulate within mitochondria depending on their physicochemical properties and mitochondrial membrane potential [41].

In the present study, MitoTracker staining showed that PUE-ZnO NPs were well tolerated by HaCaT keratinocytes, with only minor changes in mitochondrial morphology observed at the highest tested concentration. In contrast, A375 melanoma cells exhibited concentration-dependent alterations in mitochondrial staining pattern and organization, which became progressively more evident at higher concentrations. These changes are consistent with the reduced viability, morphological alterations, and nuclear damage observed in A375 cells, suggesting that mitochondrial involvement may contribute to the cytotoxic response induced by PUE-ZnO NPs. However, further assays evaluating mitochondrial membrane potential, reactive oxygen species generation, or apoptosis-related markers would be required to confirm the exact mechanism.

Given the cytotoxic effects observed following PUE-ZnO NP treatment, a colony formation assay was performed to determine whether these effects extend beyond the initial exposure period and influence the long-term proliferative capacity of A375 melanoma cells and HaCaT keratinocytes. The clonogenic assay is considered important for assessing the long-term efficacy of treatment, particularly important in cancer therapy, where preventing tumor cell repopulation and sustained proliferation is a major therapeutic strategy [42]. In HaCaT cells, treatment with PUE-ZnO NPs resulted in a reduction in colony-forming capacity of up to 60.03% at the highest concentration (200 μg/mL). Meanwhile, at lower concentrations, colony-forming capacity remained above 70%. In contrast, in A375 melanoma cells, a considerable area-dependent reduction was observed, reaching 12.26% at the highest concentration tested.

These findings indicate that PUE-ZnO NPs markedly impair the long-term proliferative potential of A375 melanoma cells, especially at higher concentrations. Importantly, the reduction in colony formation was substantially stronger in A375 cells than in HaCaT cells, supporting the preferential antimelanoma effect suggested by the MTT, morphological, nuclear, and mitochondrial evaluations. However, the decrease observed in HaCaT cells at the highest concentrations also indicates that the long-term response of keratinocytes should be considered when defining an optimal therapeutic concentration range.

Overall, the comparative evaluation of HaCaT keratinocytes and A375 melanoma cells indicate that PUE-ZnO NPs exert a stronger biological effect on melanoma cells than on non-tumorigenic keratinocytes. In HaCaT cells, the formulation was generally well tolerated in the short-term viability assay, with only moderate reductions in clonogenic capacity at the highest concentrations. In contrast, A375 cells displayed a marked decrease in metabolic activity, evident morphological alterations, nuclear changes suggestive of apoptosis, mitochondrial staining alterations, and a strong reduction in colony-forming ability. The agreement between these complementary assays supports the hypothesis that PUE-ZnO NPs display a preferential antimelanoma effect under the tested in vitro conditions. The stronger response observed in A375 cells may be associated with the known susceptibility of cancer cells to ZnO NPs-induced stress. Previous studies have reported that ZnO nanoparticles can induce cytotoxicity in tumor cells through mechanisms involving oxidative stress, mitochondrial alterations, DNA damage, and apoptosis [43,44].

However, the present study represents a preliminary screening of the antimelanoma potential of PUE-ZnO NPs and has several limitations that should be addressed in future investigations. First, the biological evaluation was performed using a single 2D human melanoma cell line, therefore, additional studies should include other melanoma cell lines with distinct molecular characteristics, as well as more physiologically relevant 3D models, such as spheroids, organoids, or reconstructed tissues. Furthermore, the biological effects were evaluated only after 24 h of exposure. Future studies should investigate longer treatment periods (48 and 72 h) and extend the concentration range to better characterize the time and dose-dependent responses.

Although the observed cytotoxic effects were accompanied by alterations in cellular morphology, mitochondrial membrane potential, and oxidative stress, the precise molecular mechanisms underlying these effects remain to be elucidated. Future studies should investigate the molecular pathways involved in these effects by assessing apoptosis-specific markers and assays, including Annexin V/PI staining, caspase activation, cytochrome c release, and the expression of apoptosis-related proteins such as BAX and BCL-2. Furthermore, the role of ROS generation and apoptosis-related signaling pathways should be elucidated to better define the mechanisms responsible for the observed cytotoxicity. Finally, after identifying the optimal formulation and route of administration, the antitumor efficacy and safety of PUE-ZnO NPs should be validated in appropriate in vivo models before considering their translational potential.

## 5. Conclusions

In conclusion, PUE-ZnO NPs showed a preferential cytotoxic effect against A375 melanoma cells while maintaining better compatibility with HaCaT keratinocytes. The nanoparticles largely preserved the long-term proliferative capacity of HaCaT cells at low and intermediate concentrations, whereas they dose-dependently reduced the clonogenic capacity of A375 melanoma cells, particularly at higher concentrations. These results support the potential of PUE-ZnO NPs as an in vitro antimelanoma candidate, but further mechanistic studies are needed to confirm the pathways involved and to define an optimal concentration range with minimal effects on non-tumorigenic skin cells.

## Data Availability

The original contributions presented in this study are included in the article/Supplementary Material. Further inquiries can be directed to the corresponding authors.

## Supplementary Materials

The following supporting information can be downloaded at https://www.mdpi.com/article/10.3390/jox16040146/s1. The original, uncropped images used in manuscript are provided in the following Supplementary Files: (i) Supplementary File S1 corresponding to Figure 2A; (ii) Supplementary File S2 corresponding to Figure 2B; (iii) Supplementary File S3 corresponding to Figure 3; (iv) Supplementary File S4 corresponding to Figure 4; (v) Supplementary File S5 corresponding to Figure 5; (vi) Supplementary File S6 corresponding to Figure 6.
